# Supplementary material for: Chromothripsis during telomere crisis is independent of NHEJ, and consistent with a replicative origin
Source: Genome Res. 2019 May;29(5):737–49. doi: 10.1101/gr.240705.118 (PMC6499312; doi:10.1101/gr.240705.118)
Supplement: Supplemental Material [file supp_gr.240705.118_Supplemental_file_1.zip › contigs/annotated_contigs/DB93/contig.2.DB93_length_670_mean_cov_6.92835820896.docx]

**DB93_length_670_mean_cov_6.92835820896**

```
TTATCTTATGTAGGATGGGATCTCACTGTGTAGTCCAGGCTGGGCTCAAACTCCTGACCTCAAGTGATCATCCTGCCTTGGTCTCCCAA
 >chr17:57807798-57808114 + E=2e-178 p=3e-02
AGTGCTGGGATTATAGGCATGAGCCACTGCACCCGGCCCCAGTTGTCTTTTTATAAGTGTCTGGGCAAGTTTTAATATTGTATTAAGGA

CTCAGCATTTATTGTATTAAGGACTCAGCATTTTAGCCTTTTATTGAAGTGATGTGCTTTTTGAGTAGAAAGTTTTGCTTTACTAACAC

CTGAAGAATGATACAATCACAGGCAATCTGGTTTTTATATGTTGTATTA|T|ATGATGACTTTTATTAATATGGATGATTTAAGTAGAA
                                                   >chr17:57809373-57809715 + E=6e-179
ACTTTCCTTCATGCATTTAAAGGAAAATGAAAAAAAAGTTTTTCTTTTGTTGTAATCATACTTTTTTTTTAGCTTTTGAATTGTTTGAC

ATCTCAAACACATGGCAAATAATACTCTAGTGTGGTAATATTTTCACAGCAATCCTCTACTTTTAGAGGAAGTGCAAGGAATCTGAAAT

AGTAGTTTTCATTTGTTTGTTTGTTTGTTTGCTTTTTGAGACGGAATCTCACTCTGTCACCAGGCTCGAGTGCAATGGTGCGATCTTGG

CTCACTGCAACCTCCGCCTCCCGCGTTCAAGCGATTCT|CCTGCCTCAGC
```
